# Multi-omic profiling converges on proteasome subunits PSMA7/PSMB2 as targets of the sepsis-protective agent Handelin

**DOI:** 10.3389/fimmu.2026.1782122

**Published:** 2026-03-19

**Authors:** Dexiu Chen, Qian Zhang, Yuanxin Wu, Yingchun Hu, Muhu Chen

**Affiliations:** 1 Department of Critical Care Medicine, The Affiliated Hospital, Southwest Medical University, Luzhou, Sichuan, China; 2 Department of Infectious Diseases Department, The Affiliated Hospital, Southwest Medical University, Luzhou, Sichuan, China; 3 Department of Emergency Department, The Affiliated Hospital, Southwest Medical University, Luzhou, Sichuan, China; 4 Skin Structure and Function Key Laboratory of Luzhou, Department of Dermatology, The Affiliated Hospital, Southwest Medical University, Luzhou, Sichuan, China

**Keywords:** sepsis, 26S proteasome, DIA-CETSA, Handelin, PSMA7, PSMB2

## Abstract

**Introduction:**

Sepsis is a life-threatening systemic inflammatory syndrome with limited targeted therapeutic options. Handelin, a natural compound derived from Chrysanthemum indicum, exhibits anti-inflammatory activity, yet its direct targets and protective mechanisms in sepsis remain unclear.

**Methods:**

We established a lipopolysaccharide (LPS)-induced sepsis-like model in zebrafish larvae to evaluate the protective effects of Handelin. Survival, locomotor behavior, macrophage recruitment, and systemic reactive oxygen species (ROS) levels were assessed. To identify direct protein targets, we performed drug affinity responsive target stability profiling using data-independent acquisition mass spectrometry (DIA-CETSA) in macrophages. Clinical relevance was examined via 4D-DIA proteomics of plasma from a sepsis patient cohort and meta-analysis of public transcriptomic datasets. Molecular docking and dynamics simulations were used to characterize binding interactions.

**Results:**

Handelin significantly improved survival, restored locomotor activity, suppressed macrophage aggregation, and reduced ROS in zebrafish. DIA-CETSA revealed that Handelin specifically stabilized multiple core subunits of the 26S proteasome, most notably PSMA7 and PSMB2. In sepsis patients, higher plasma levels of PSMA7 and PSMB2 were associated with increased 90-day mortality and positively correlated with markers of liver injury and SOFA scores. Transcriptomic meta-analysis across 10 independent cohorts revealed that PSMA7 expression was significantly higher in non-survivors than survivors, while PSMB2 showed a similar trend that was marginally significant and sensitive to cohort composition. These findings highlight the complex relationship between transcriptional regulation and clinical outcomes in sepsis, with favorable predicted binding affinities.

**Discussion:**

This study suggests that the protective effect of Handelin in sepsis may be associated with its stabilization of the core proteasome subunits PSMA7 and PSMB2. These findings provide new pharmacological insights into the potential anti-septic application of Handelin and propose a novel strategic direction for the treatment of sepsis through precise modulation of proteasome function.

## 1 Introduction

Sepsis, a systemic inflammatory response syndrome triggered by infection, represents a leading cause of global mortality, with a burden that is particularly severe in low- and middle-income countries (1). Its core pathological process involves the excessive activation and dysregulation of the immune system, ultimately leading to life-threatening multiple organ dysfunction (2). Despite continuous advances in supportive clinical care, the development of targeted therapeutics directed against its central inflammatory pathways has not yielded significant breakthroughs (3). Therefore, the continued exploration of novel lead compounds from natural products that can modulate the innate immune response, coupled with the elucidation of their precise mechanisms of action, holds significant value for innovating sepsis treatment strategies.

Handelin, a dimeric sesquiterpene lactone (4), is a key anti-inflammatory constituent derived from the traditional medicinal herb Chrysanthemum indicum (5). Substantial prior research has demonstrated that Handelin exhibits broad anti-inflammatory and cytoprotective activities across various cellular and animal models. It effectively inhibits lipopolysaccharide (LPS)-induced activation of the NF-κB and MAPK signaling pathways and the subsequent production of pro-inflammatory mediators in macrophages. Furthermore, it ameliorates pathological phenotypes in diverse disease models, including pulmonary emphysema and muscle atrophy, with its known actions implicating several potential targets such as Hsp70 and TAK1 (6–8). However, the *in vivo* role and precise molecular mechanism of Handelin in the context of sepsis remain undefined. In the present study, using an LPS-induced sepsis-like model in zebrafish larvae, we found that Handelin completely reversed the lethal effect, rescued locomotor dysfunction, and significantly suppressed aberrant macrophage activation and systemic oxidative stress. This robust, multi-faceted protective phenotype suggests that Handelin may act upon a critical upstream regulatory node within the inflammatory signaling network.

To identify the direct molecular target(s) of Handelin, we employed the data-independent acquisition–cellular thermal shift assay (DIA-CETSA) technique for global proteomic analysis (9). Initial profiling revealed that Handelin specifically binds to and significantly enhances the thermal stability of multiple core subunits of the 26S proteasome, including PSMA7 and PSMB2. Notably, the degradation of IκBα, a key inhibitory protein of the NF-κB pathway, is precisely dependent on the 26S proteasome (10), hinting at a potential mechanistic link. Further analysis of a clinical cohort revealed that higher expression of PSMA7 and PSMB2 in sepsis patients was associated with favorable survival outcomes, yet also correlated with higher disease severity scores. This suggests that these proteasome subunits may play a complex and dynamic regulatory role during inflammatory stress.

Based on these findings, we propose the central hypothesis of this study: Handelin may exert its core anti-septic mechanism by directly targeting and binding to core proteasome subunits (e.g., PSMA7/PSMB2), thereby uniquely modulating their functional state and subsequently interfering with the excessive activation of critical inflammatory signaling pathways such as NF-κB. To test this hypothesis, our study integrates multi-tiered evidence from chemoproteomic target screening, clinical cohort correlation analysis, and computational chemical simulations, aiming to construct a coherent evidence chain from direct target identification to clinical relevance. This work not only provides novel mechanistic insights supporting the development of Handelin as an anti-sepsis lead compound but also opens new perspectives for understanding the complex functions of the proteasome within the inflammatory immune microenvironment.

## 2 Materials and methods

### 2.1 Clinical sample collection and ethics statement

This study prospectively collected peripheral blood samples from 43 adult sepsis patients and 9 age-matched healthy controls between July and October 2025 in the Intensive Care Unit of the Affiliated Hospital of Southwest Medical University. Sepsis was diagnosed according to the SEPSIS-3.0 criteria (confirmed or suspected infection plus a Sequential Organ Failure Assessment score ≥2). For sepsis patients, blood samples were drawn within 24 hours of ICU admission to ensure a standardized collection window relative to the clinical care pathway. It should be noted that patients were enrolled at various stages of their disease course upon ICU admission; therefore, the timing relative to sepsis onset may vary. Healthy control samples were collected upon enrollment. Plasma was separated from whole blood by centrifugation and immediately stored at -80 °C until analysis. Key exclusion criteria were: age <18 years, pre-existing end-stage organ failure, pregnancy, or patient/family refusal to participate. Patients who had received immunomodulatory therapies (including systemic corticosteroids, cytokine antagonists, or other biologics) were not excluded *a priori*; however, a comprehensive review of medical records confirmed that none of the enrolled sepsis patients had received such treatments within one week prior to blood sampling.

The study was conducted in accordance with the principles of the Declaration of Helsinki and was approved by the Ethics Committee of the Affiliated Hospital of Southwest Medical University (Approval No. KY2025-390). Written informed consent was obtained from all participants or their legal representatives.

### 2.2 Establishment of a sepsis-like model in zebrafish and phenotypic analysis

#### 2.2.1 Zebrafish husbandry and embryo collection

Adult AB strain zebrafish were maintained in a recirculating system at 28°C under a 14-hour light/10-hour dark cycle and fed three times daily. To obtain embryos, male and female fish were paired at a 1:1 ratio in breeding tanks separated by a divider the night before spawning. The divider was removed at the onset of light the next morning, and embryos spawned within one hour were collected. Embryos were incubated in 1× E3 embryo medium (5 mM NaCl, 0.17 mM KCl, 0.33 mM CaCl_2_, 0.33 mM MgSO_4_, pH 7.2; all inorganic salts were analytical grade from Sinopharm Chemical Reagent Co., Ltd.) containing 0.3 ppm methylene blue (Sigma-Aldrich, M4159). They were raised at 28.5°C in an incubator (Ruiju, RGX-70ES) under the same light cycle as adults.

#### 2.2.2 LPS-induced sepsis-like model and drug treatment

To evaluate the effects of Handelin on inflammation-associated phenotypes without inducing significant lethality, a sublethal concentration of LPS was used. At 6 hours post-fertilization (hpf), normally developing embryos were selected for experiments. Embryos were randomly allocated into 6-well cell culture plates (Corning, 3516), with 10 embryos per well and 3 biological replicate wells per group. The groups and treatments were as follows: (1) Control group: E3 medium only; (2) LPS model group: E3 medium containing 8 µg/mL LPS (11) (from E. coli O111:B4, Solarbio, L8880); (3) Handelin intervention group: E3 medium containing 8 µg/mL LPS and 10 µg/mL Handelin (GLPBIO, GC-46865). The treatment medium (E3 containing LPS with or without Handelinn) was renewed completely every 24 hours to maintain consistent drug and LPS exposure throughout the experimental period. This daily renewal ensures that the nominal concentrations of Handelin (10 µg/mL) and LPS (8 µg/mL) are restored, compensating for any potential degradation or uptake by the larvae.

#### 2.2.3 Survival analysis

To assess the protective effect of Handelin against lethal inflammatory stress, a survival assay was performed using the predetermined 72 hpf median lethal concentration (LC50) of LPS. At 6 hpf, embryos were randomly assigned to groups (total n=30 per group, distributed across 3 replicate wells) and treated as follows: (1) Control (E3 medium); (2) LPS model (47.7 µg/mL LPS); (3) Handelin intervention (47.7 µg/mL LPS + 10 µg/mL Handelin). The LPS concentration of 47.7 µg/mL was predetermined as the 72 hpf median lethal concentration in pilot experiments. From the start of treatment, mortalities were recorded daily until 72 hpf. The treatment medium (E3 containing LPS with or without Handelin) was renewed completely every 24 hours to maintain consistent drug and LPS exposure throughout the experimental period. This daily renewal ensures that the nominal concentrations of Handelin (10 µg/mL) and LPS (47.7 µg/mL) are restored, compensating for any potential degradation or uptake by the larvae. Kaplan-Meier survival curves were plotted using GraphPad Prism 8.0 software, and statistical differences between groups were analyzed using the log-rank test.

#### 2.2.4 Macrophage aggregation observation

To evaluate innate immune cell recruitment, 10 larvae per group were randomly selected at 72 hpf and stained with Neutral Red (Sigma-Aldrich, N2889; 5 µg/mL in E3 medium) for 2 hours at 28°C in the dark. After staining, larvae were washed, anesthetized with MS-222 (0.16 mg/mL), and mounted in 3% methylcellulose for imaging. Images were captured under a stereo fluorescence microscope (Mingmei, MZX81) with fixed parameters (exposure: 200 ms; gain: 1.5×; magnification: 40×). For unbiased quantification, images were coded with random alphanumeric identifiers by an independent researcher prior to analysis. A separate researcher, blinded to group allocation, performed quantification using ImageJ. Regions of interest (ROIs) were defined anatomically: (1) abdominal region: a 500 × 300 pixel rectangle positioned at the posterior margin of the yolk sac extension; (2) brain region: a 400 × 250 pixel rectangle dorsal to the eyes covering the optic tectum and hindbrain. Mean fluorescence intensity within combined ROIs was measured after background subtraction (rolling ball algorithm, radius: 50 pixels). Data were normalized to the control group mean.

#### 2.2.5 Locomotor behavioral analysis

To assess neuromuscular function, the treatment was extended to 144 hpf. At this timepoint, 8 larvae were randomly selected from each group and placed individually into wells of a 48-well plate (JET BIOFIL, TCP011048). After a 10-minute acclimatization period in the Zebralab high-throughput behavioral analysis system (ViewPoint Life Sciences), the autonomous swimming trajectories of larvae were automatically recorded for 30 minutes. The analysis software directly output the total distance moved and the average velocity for each larva.

#### 2.2.6 Reactive oxygen species level detection

To assess systemic oxidative stress, 10 larvae per group were randomly selected at 72 hpf and incubated with 20 µM DCFH-DA (Beyotime, S0033S) in E3 medium for 1 hour at 28°C in the dark. After incubation, larvae were washed, anesthetized with MS-222 (0.16 mg/mL), and mounted in 3% methylcellulose. Whole-body fluorescence images were captured (excitation: 488 nm; emission: 525 nm; exposure: 150 ms; gain: 1.0×; magnification: 20×). Images were coded with random alphanumeric identifiers by an independent researcher, and blinded quantification was performed using ImageJ. The ROI was defined as the entire larval body, manually outlined from snout to tail tip excluding the yolk sac. Mean fluorescence intensity was measured after background subtraction (rolling ball algorithm, radius: 100 pixels). Data were normalized to the control group mean.

### 2.3 DIA-CETSA-based screening for direct targets of Handelin

#### 2.3.1 Cell culture and treatment

Mouse RAW 264.7 macrophages were cultured in DMEM high-glucose complete medium (Gibco, C11995500BT) supplemented with 10% fetal bovine serum (Ausbian, VS500T) and 1% penicillin-streptomycin (Beyotime, C0222) at 37°C in a 5% CO_2_ incubator. Experiments were performed when cells reached 80-90% confluence. Cells were randomly divided into two groups: (1) LPS model group: stimulated with 800 ng/mL LPS for 4 hours; (2) LPS + Handelin treatment group: stimulated with 800 ng/mL LPS for 4 hours, then gently washed twice with pre-warmed PBS (Gibco, C10010500BT) and further incubated for 2 hours in fresh complete medium containing 10 µM Handelin. Each group consists of 3 independent biological replicate samples.

#### 2.3.2 Cell suspension preparation and thermal denaturation

After treatment, cells were washed twice with ice-cold PBS containing 1× protease inhibitor cocktail (Roche, 04693132001) and resuspended in the same buffer to a density of 2×10^7^ cells/mL. The cell suspension was aliquoted into multiple 0.2 mL PCR tubes. To generate a thermal stability curve, samples were subjected to a temperature gradient (37°C, 50°C, 53°C, 56°C, 59°C, 62°C, 65°C) for 3 minutes each in a precise metal bath (Bioer, LC-16), followed immediately by 3 minutes of cooling on ice to terminate the reaction. The difference in total soluble protein amount between the two groups at each temperature point was initially assessed via simple protein quantification (BCA assay). The results indicated that the difference in protein solubility was most pronounced at 65°C. This temperature was selected for the subsequent large-scale DIA-CETSA experiment, as it was deemed optimal for maximizing the enrichment of proteins stabilized by drug-target binding, thereby facilitating the unbiased discovery of potential targets in the full-proteome DIA analysis. Accordingly, the 65°C-treated samples were compared with the 37°C controls in the formal DIA-CETSA procedure.

#### 2.3.3 Protein extraction and quantification

Thermally treated cell samples were flash-frozen in liquid nitrogen and then transferred to 1.5 mL centrifuge tubes containing 100 µL of SDT lysis buffer (4% SDS, 100 mM Tris-HCl, pH 7.6). Samples were sonicated on ice using an ultrasonic homogenizer (Scientz, SCIENTZ-IID) (20% power, 2 s on/3 s off, total 2 minutes). Subsequently, samples were heated at 95°C for 5 minutes and centrifuged at 4°C, 14,000 g for 15 minutes. The supernatant was collected, and protein concentration was determined using the BCA Protein Assay Kit (Thermo Scientific, 23225).

#### 2.3.4 Protein digestion and peptide desalting

Equal amounts of protein (100 µg) were reduced with 10 mM dithiothreitol (DTT, Sigma, 43815) at 56°C for 30 minutes and alkylated with 20 mM iodoacetamide (IAA, Sigma, I1149) at room temperature in the dark for 30 minutes. Proteins were precipitated overnight by adding pre-chilled acetone. The precipitated protein pellets were redissolved in 100 mM triethylammonium bicarbonate (TEAB, Sigma, T7408) and digested overnight at 37°C with sequencing-grade trypsin (Promega, V5113) at an enzyme-to-substrate ratio of 1:50 (w/w). The resulting peptides were desalted using C18 StageTips, dried by vacuum centrifugation, and reconstituted in 0.1% formic acid.

#### 2.3.5 DIA mass spectrometry acquisition

Peptide samples were analyzed using a nano-flow liquid chromatography system (Thermo Scientific, UltiMate 3000 RSLChano) coupled to an Orbitrap Astral mass spectrometer (Thermo Scientific). Chromatographic separation was performed on an Acclaim PepMap 100 C18 column (75 µm × 25 cm, Thermo Scientific). The mass spectrometer was operated in DIA mode. Full MS scans were acquired from 350 to 1200 m/z at a resolution of 120,000. DIA scans were performed using variable windows, with a total of 40 windows whose isolation width was dynamically adjusted based on ion density.

#### 2.3.6 DIA data analysis

Raw mass spectrometry data were processed and searched using Spectronaut software (version 19.0, Biognosys). The mouse protein reference database was downloaded from UniProt (Proteome ID UP000000589, release December 2024). Primary search parameters were: trypsin digestion with up to 2 missed cleavages; fixed modification of cysteine carbamidomethylation; variable modifications of methionine oxidation and protein N-terminal acetylation. Protein and peptide identification were controlled at a 1% false discovery rate (FDR). Relative protein quantification was based on the peak area of fragment ion chromatograms extracted in DIA mode.

For quantitative analysis, all three biological replicates from each group (LPS model and LPS + Handelin) were retained. Protein abundances were normalized using the median normalization method embedded in Spectronaut to correct for systematic differences between runs. The quantitative reproducibility among replicates was assessed by calculating the median coefficient of variation (CV) of protein abundances within each group; the median CV was <15%, indicating high technical reproducibility. Thermal stability changes induced by Handelin were represented as the log_2_ ratio (log_2_FC) of normalized protein abundance in the LPS + Handelin group versus the LPS-only group at 65°C. A moderated t-test implemented via the limma R package was applied to account for the small sample size and to provide robust variance estimation. Multiple testing correction was performed using the Benjamini–Hochberg method to control the false discovery rate. Proteins with log_2_ fold change (log_2_FC) > 1 and adjusted P-value (*P*-adj) < 0.05 were considered significantly stabilized (positive log_2_FC) by Handelin. A total of 437 proteins met this criterion and were subjected to subsequent functional enrichment analysis.

### 2.4 4D-DIA proteomic analysis of clinical plasma samples

#### 2.4.1 Protein extraction and quantification

Plasma samples were lysed on ice for 30 minutes in RIPA lysis buffer (Beyotime, P0013B) containing 1 mM PMSF (Beyotime, ST506) and 1× phosphatase inhibitor cocktail (Beyotime, P1082). Lysates were centrifuged at 4°C, 12,000 g for 15 minutes, and the supernatant was collected. Protein concentration was determined using the BCA Protein Assay Kit (Thermo Scientific, 23225). After normalization, equal amounts of protein from each sample were subjected to SDS-PAGE to verify integrity.

#### 2.4.2 Peptide preparation

Equal amounts of plasma proteins were digested using the SP3 bead-based method. Briefly, samples were mixed with carboxylate-modified magnetic beads (Cytiva, 45152105050250 and 65152105050250), and acetonitrile was added to a final concentration of approximately 70% to induce protein binding. After binding, beads were washed with 70% ethanol and acetonitrile. Proteins on beads were reduced with 10 mM DTT and alkylated with 50 mM IAA. Digestion was performed overnight at 37°C with Trypsin protease (Specific Sciences, YJ-O-068-100) and LysC (Wako, 125-05061). Peptides were desalted using a C18 column (Thermo Scientific, 60109-401), dried by vacuum centrifugation, and reconstituted in 0.1% formic acid.

#### 2.4.3 4D-DIA mass spectrometry data acquisition

Peptide samples were separated using a nanoElute nano-flow LC system (Bruker Daltonics) and analyzed on a timsTOF Pro 2 mass spectrometer (Bruker Daltonics). A 90-minute chromatographic gradient was used. The mass spectrometer was operated in parallel accumulation-serial fragmentation (PASEF) DIA mode. The ion mobility scan range was set from 1/K_0_ = 0.7 to 1.45 V·s/cm². Each MS1 scan was followed by 10 consecutive DIA windows, dynamically adjusted based on ion mobility. All samples were spiked with iRT standard peptides (Biognosys, Ki-3002-2) prior to injection for retention time calibration.

#### 2.4.4 Data processing and protein identification/quantification

Raw data files (.d format) were processed using Spectronaut Pulsar software (version 19.3, Biognosys). The search database was the UniProt Human Reference Proteome (Proteome ID UP000005640, release February 7, 2025). Search parameters were similar to those described in section 2.3.6. Protein quantification was based on the peak area of ions extracted in DIA mode.

### 2.5 Bioinformatics and statistical analysis

#### 2.5.1 Functional enrichment analysis

Proteins with significantly altered thermal stability from the DIA-CETSA screen (defined as |log_2_FC| > 1 and *P*-adj < 0.05) were subjected to systematic functional annotation. Gene Ontology (GO) and Kyoto Encyclopedia of Genes and Genomes (KEGG) pathway enrichment analyses were performed using the clusterProfiler R package. A significance threshold of FDR < 0.05 was applied.

#### 2.5.2 Gene set enrichment analysis

To evaluate the global impact of Handelin treatment on biological pathways, GSEA was performed. Using the fgsea R package, a pre-ranked GSEA was conducted on the classic pathway gene sets (c2.cp) from the MSigDB database, using the thermal stability change values (log_2_FC) of all quantifiable proteins as the ranked list. Significance was judged by a normalized enrichment score (|NES| > 1.5) and FDR < 0.05.

#### 2.5.3 Clinical data analysis: survival and correlation

##### 2.5.3.1 Survival analysis

Sepsis patients were stratified into high- and low-expression groups based on the median plasma protein expression levels of PSMA7 and PSMB2. The 90-day survival rates were compared using the Kaplan-Meier method and the Log-rank test.

##### 2.5.3.2 Correlation analysis

The correlation between continuous clinical indicators and target protein expression was assessed using Pearson correlation coefficient or Spearman’s rank correlation coefficient, depending on data distribution. P-values were adjusted for multiple comparisons using the Benjamini-Hochberg method. A corrected *P*-adj < 0.05 was considered statistically significant.

#### 2.5.4 Multi-group comparisons

For comparisons of protein/mRNA expression among multiple groups (e.g., healthy control, survivor, non-survivor), normality and homogeneity of variances were first tested. If assumptions were met, one-way ANOVA followed by appropriate *post-hoc* tests was used; otherwise, the Kruskal-Wallis test followed by Dunn’s *post-hoc* test was applied. The significance level was set at α = 0.05.

#### 2.5.5 Meta-analysis of public transcriptomic data

Sepsis-related transcriptomic datasets were systematically retrieved from the Gene Expression Omnibus (GEO) database. Ten independent datasets (total n=1,174 samples: 307 healthy controls and 867 sepsis patients) were included. Meta-analyses were performed using the meta R package. The standardized mean difference (SMD) with 95% confidence interval (CI) was used as the effect size. Heterogeneity across studies was assessed using the I² statistic, τ², and Cochran’s Q test. I² values of 25%, 50%, and 75% were considered low, moderate, and high heterogeneity, respectively. Due to the anticipated clinical and methodological heterogeneity, a random-effects model (DerSimonian–Laird method) was applied for all analyses to provide more conservative estimates. To assess the influence of individual studies on the pooled estimate, leave-one-out sensitivity analysis was performed by iteratively removing one study at a time and recalculating the overall effect size. Potential publication bias was examined by visual inspection of funnel plots and quantified using Egger’s linear regression test. A *P*-value < 0.05 was considered indicative of significant asymmetry. For comparisons with fewer than 10 studies, Egger’s test results were interpreted with caution due to limited power.

### 2.6 Molecular docking and dynamics simulations

The crystal structure of the 26S proteasome containing the PSMA7 and PSMB2 subunits (PDB ID: 9HMN) was obtained from the Protein Data Bank. Receptor proteins were prepared for docking (addition of hydrogens, assignment of charges) using AutoDock Tools 3 (12, 13). The 3D structure of Handelin was generated using Open Babel. Docking pocket selection was based on the distinct functional roles of the target subunits (14). For PSMB2, which harbors the trypsin-like (T-L) proteolytic activity, the docking grid was centered on the known catalytic site, with specific focus on Thr53—a residue adjacent to the catalytic N-terminal threonine (Thr1)—to assess potential interactions with the active center. For PSMA7, which lacks intrinsic catalytic activity and serves primarily regulatory functions in gate-keeping, potential ligand-binding pockets were predicted using the PocketFinder algorithm within AutoDock Tools and the CASTp server. The highest-ranked pocket, located at the intersubunit interface of the α-ring and comprising residues Gln54, Arg60, Ile62, Val59, and Ile84, was selected for docking. This pocket is proximal to the α-ring gate region that controls substrate entry into the 20S core particle. This combined strategy—targeting both known functional sites (PSMB2 catalytic center) and predicted allosteric cavities (PSMA7 regulatory pocket)—was designed to comprehensively explore possible binding modes of Handelin.

Semi-flexible docking was performed using AutoDock Vina, with a grid box defined to cover the selected pockets as described above. The conformation with the most favorable predicted binding free energy was selected for analysis. Subsequently, molecular dynamics simulations were performed using Desmond software. The docked complex was solvated in a cubic box of TIP3P water molecules and neutralized with 0.15 M NaCl. After energy minimization and stepwise equilibration, a 100 ns production simulation was conducted in the NPT ensemble (300 K, 1 bar). Trajectory coordinates were saved every 100 ps. The stability of the complex was assessed by analyzing protein backbone root-mean-square deviation (RMSD) and ligand-protein interaction frequencies.

## 3 Results

### 3.1 Handelin confers comprehensive protection in a zebrafish sepsis-like model

**Figure 1:**
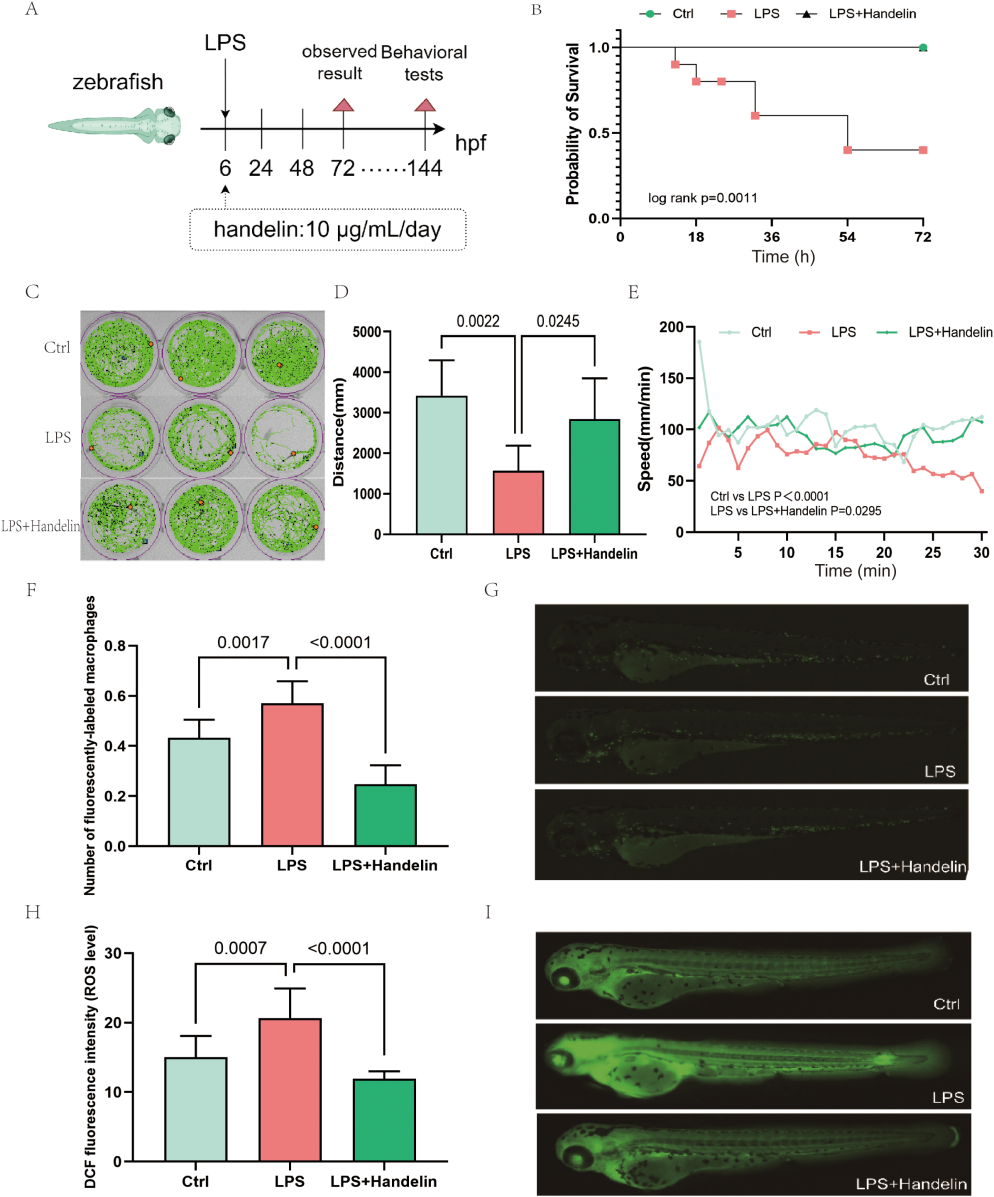
Handelin alleviates LPS-induced systemic inflammation and improves survival and behavioral performance in zebrafish larvae. **(A)** Schematic illustration of the experimental design. Zebrafish larvae at 6 hpf were co-treated with LPS (8 µg/mL for phenotypic assays or 47.7 µg/mL for survival assay) and Handelin (10 µg/mL). The treatment medium was renewed daily. Phenotypic analyses (macrophage aggregation, ROS levels, locomotor behavior) were performed at 72 or 144 hpf as indicated. **(B)** Survival curves of zebrafish larvae exposed to LPS with or without Handelin treatment(Log-rank test). **(C)** Representative trajectories of zebrafish larval movement during a 30-minute behavioral test at 144 hpf. **(D)** Quantification of the total movement distance of larvae during the 30-minute test.Data are presented as mean ± SEM. One-way ANOVA with Tukey’s *post hoc* test was performed. **(E)** Time-course changes in the average movement speed of larvae during the 30-minute test.Data are presented as mean ± SEM. **(F)** Quantitative analysis of macrophage fluorescence intensity in zebrafish larvae at 72 hpf. Data are presented as mean ± SEM. One-way ANOVA with Tukey’s *post hoc* test was performed. **(G)**Representative fluorescent images showing macrophage distribution(indicated by arrows)in different treatment groups at 72 hpf. Scale bar,200μm. **(H)** Quantitative analysis of relative ROS levels in zebrafish larvae at 72 hpf,as measured by DCFH-DA fluorescence intensity. Data are presented as mean ± SEM. One-way ANOVA with Tukey’s *post hoc* test was performed. **(I)** Representative fluorescent images showing ROS levels in different treatment groups at 72 hpf.Scale bar,200μm.

To evaluate the protective effects of Handelin against systemic inflammation, we established a sepsis-like model in zebrafish larvae using LPS induction (**Figure 1A**). Survival analysis showed that stimulation with the median lethal dose of LPS significantly reduced larval survival at 72 hpf, whereas Handelin treatment completely reversed this lethal effect, restoring survival to a level statistically indistinguishable from the control group (Log-rank test, *P* = 0.0011; **Figure 1B**). Behaviorally, LPS stimulation markedly decreased the total movement distance and average swimming speed of larvae during a 30-minute test period, and Handelin effectively prevented this motor impairment (**Figures 1C–E**). Further mechanistic investigations revealed that Handelin significantly suppressed LPS-triggered innate immune responses. *In vivo*, LPS induced pronounced aggregation of macrophages in the abdominal and brain regions of zebrafish, which was clearly inhibited by Handelin (**Figures 1F, G**). Concurrently, the sharp increase in systemic reactive oxygen species (ROS) levels induced by LPS was also significantly reversed by Handelin treatment (**Figures 1H, I**). These findings indicate that Handelin exhibits potent anti-inflammatory and organ-protective activities in this *in vivo* model.

### 3.2 DIA-CETSA screening identifies Handelin specifically stabilizing core subunits of the 26S proteasome

**Figure 2:**
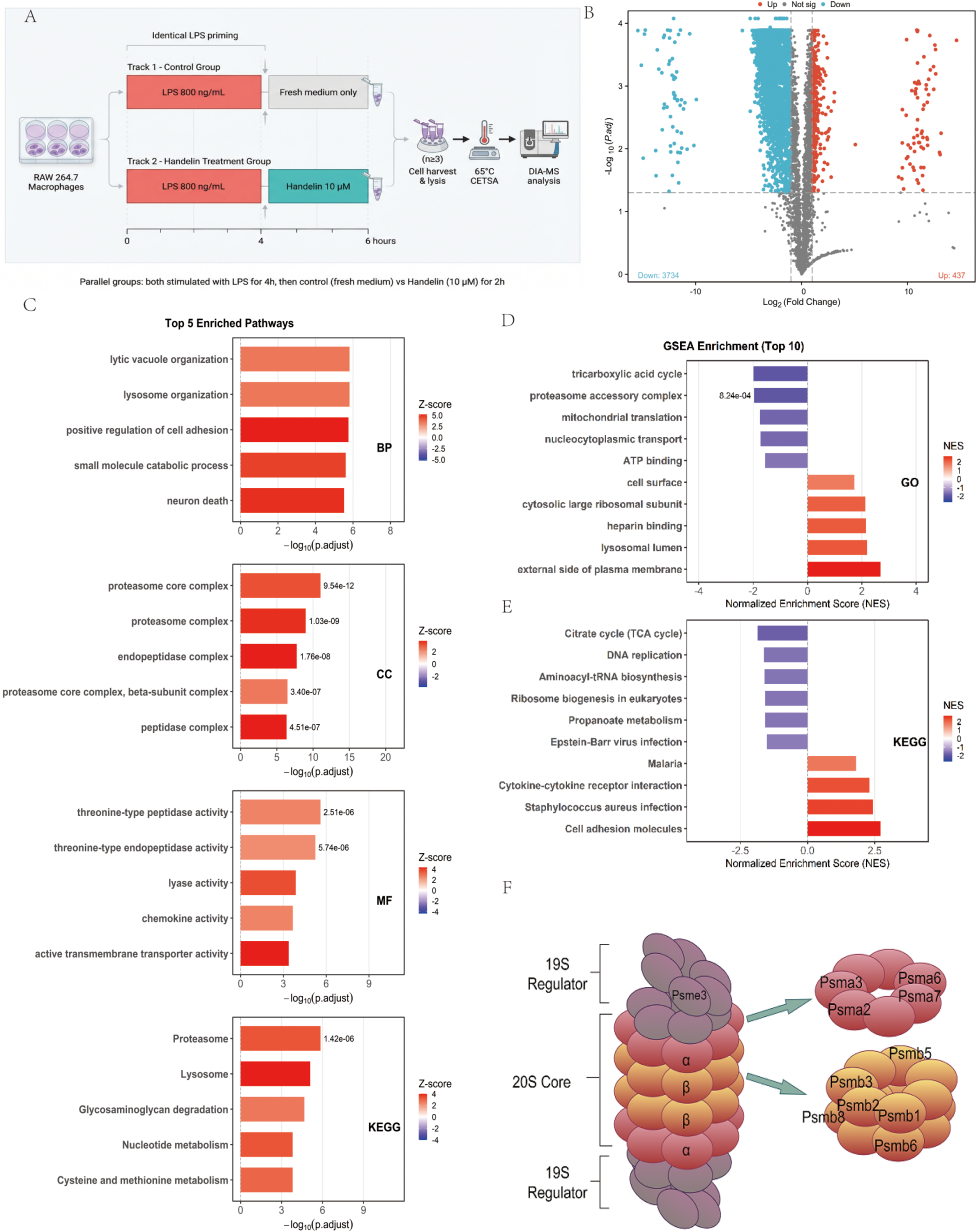
DIA-CETSA screening identifies the 26S proteasome as a primary target complex of Handelin. **(A)** Schematic workflow of the DIA-CETSA experiment in RAW 264.7 macrophages. **(B)** Volcano plot displaying proteins with significantly altered thermal stability upon Handelin treatment.Proteins with increased stability are highlighted in red. **(C)** Top GO cellular component terms and KEGG pathways enriched among the 437 proteins with increased thermal stability. **(D)** GSEA on all proteins exhibiting alterations in thermal stability. Among these, **(D)** depicts the top 10 GO enrichment terms, and **(E)** showcases the top 10 KEGG signaling pathways. **(F)** The eleven proteins exhibiting enhanced thermal stability within the 26S proteasome.

To identify direct protein targets of Handelin under conditions relevant to its potential therapeutic use, we employed a “treatment-mimicking” DIA-CETSA approach. RAW 264.7 macrophages were first stimulated with LPS for 4 hours to establish an inflammatory state (15, 16), followed by a 2-hour treatment with Handelin. This design prioritizes the detection of drug-target engagement in a pathophysiologically relevant context, rather than assessing immediate anti-inflammatory efficacy, which will be addressed in subsequent functional studies (**Figure 2A**). Compared to the LPS-only group, the LPS + Handelin group showed significantly increased thermal stability of 437 proteins (|log_2_FC| > 1, *P*–adj < 0.05) (**Figure 2B**). KEGG pathway enrichment analysis of these potential targets revealed “Proteasome” as the most significantly enriched term. Similarly, GO cellular component analysis indicated that these proteins were most prominently enriched in the “proteasome core complex” (**Figure 2C**). Gene set enrichment analysis (GSEA) of all thermally shifted proteins further confirmed significant perturbation of the “Proteasome” pathway and suggested that Handelin may affect broader immune and metabolic pathways (**Figures 2D, E**). Together, these data indicate that the 26S proteasome is a major target complex of Handelin. Specifically, we identified 11 thermally stabilized proteins belonging to the 26S proteasome, including α–subunits (PSMA2, PSMA3, PSMA6, PSMA7) and β–subunits (PSMB1, PSMB2, PSMB3, PSMB5, PSMB6, PSMB8) of the core particle, as well as the regulatory particle component PSME3 (**Figure 2F**).

### 3.3 Clinical relevance of key proteasome subunits PSMA7 and PSMB2 in sepsis patients

**Figure 3:**
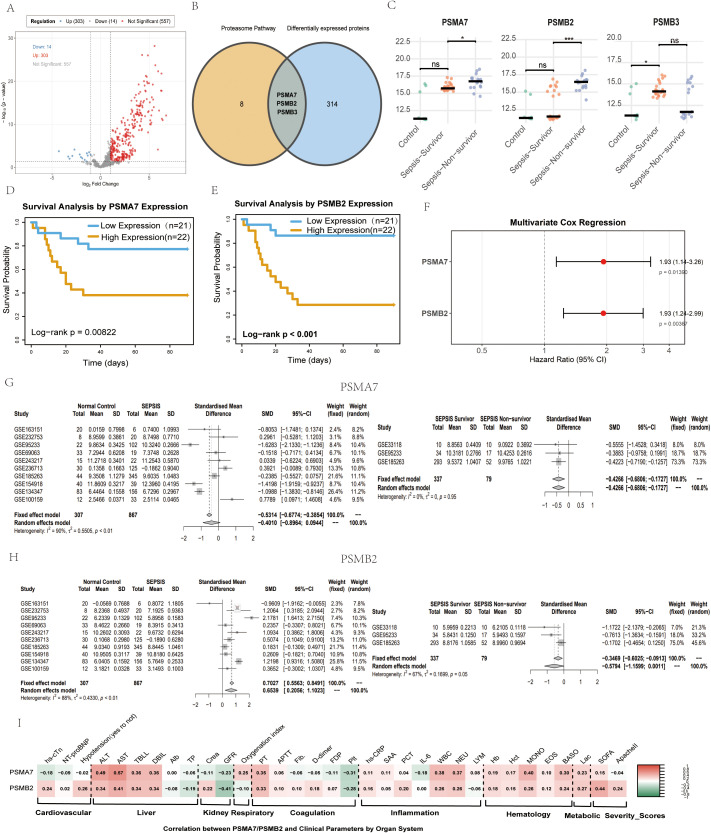
Clinical relevance of proteasome subunits PSMA7 and PSMB2 in sepsis. **(A)** Volcano plot of differentially expressed proteins between the healthy control group and the sepsis patient group based on 4D – DIA **(B)** Venn diagram showing the overlap between Handelin-stabilized proteasome subunits and differentially expressed proteins in a sepsis patient plasma cohort(4D-DIA proteomics). **(C)** The relative expression levels of PSMA7, PSMB2 and PSMB3 in the healthy control group, the survival group of sepsis and the non-survival group of sepsis. **(D-F)** Kaplan-Meier survival curves for sepsis patients stratified by high or low plasma levels of PSMA7 **(D)** PSMB2 **(E)** (Log-rank test). **(F)** Multivariate regression analysis of PSMA7 and PSMB2 after adjusting for age and SOFA score. **(G, H)** Integrated analysis of GEO transcriptomic datasets:mRNA expression levels of PSMA7 **(G)** and PSMB2 **(H)** in healthy controls,sepsis survivors,and non-survivors. **(I)** Correlation heatmap between plasma levels of PSMA7/PSMB2 and clinical severity indicators (SOFA score, AST, ALT, PT). Spearman’s correlation coefficients and p-values are indicated. * represents p < 0.05, ** represents p < 0.01, ***represents p < 0.001.

To explore the clinical relevance of the above potential Handelin targets, we collected plasma from 9 healthy controls and 43 sepsis patients for 4D–DIA proteomic sequencing (baseline characteristics in **Table 1**). Differential protein analysis (|log_2_FC| > 1, *P*–adj < 0.05) identified 303 upregulated and 14 downregulated proteins (**Figure 3A**). Intersecting the differentially expressed proteins with the 11 proteasome subunits revealed PSMA7, PSMB2, and PSMB3 as common candidates (**Figure 3B**). Comparing the relative expression levels of these three proteins among healthy controls, sepsis survivors, and non–survivors showed that PSMA7 and PSMB2 were differentially expressed between survivors and non–survivors, whereas PSMB3 expression differed significantly between controls and sepsis survivors (**Figure 3C**).

**Table 1:** Baseline clinical information.

Variable	Overall, (n = 52)^1^	Control(n = 9) (17%)^1^	Sepsis(n= 43) (83%)^1^	Statistics	p-value^2^
age	71.00 [60.00, 79.25]	66.00 [60.00, 71.00]	71.00 [61.00, 80.50]	125	0.100
Sex(1male,2female)					0.247
1	34 (65.38%)	4 (44.44%)	30 (69.77%)		
2	18 (34.62%)	5 (55.56%)	13 (30.23%)		
BMI	23.11 [20.09, 24.24]	21.36 [19.45, 23.80]	23.12 [20.49, 24.32]	161	0.439
Diabetes(1yes,2no)					0.664
1	11 (21.15%)	1 (11.11%)	10 (23.26%)		
2	41 (78.85%)	8 (88.89%)	33 (76.74%)		
Cardiovasculardisease(1yes,2no)					0.140
1	26 (50.00%)	2 (22.22%)	24 (55.81%)		
2	26 (50.00%)	7 (77.78%)	19 (44.19%)		
COPD(1yes,2no)					0.544
1	4 (7.69%)	1 (11.11%)	3 (6.98%)		
2	48 (92.31%)	8 (88.89%)	40 (93.02%)		
CKD(1yes,2no)					>0.999
1	3 (5.77%)	0 (0.00%)	3 (6.98%)		
2	49 (94.23%)	9 (100.00%)	40 (93.02%)		
Hypohepatia (1yes,2no)					>0.999
1	1 (1.92%)	0 (0.00%)	1 (2.33%)		
2	51 (98.08%)	9 (100.00%)	42 (97.67%)		
Autoimmunedisease (1yes,2no)					>0.999
1	1 (1.92%)	0 (0.00%)	1 (2.33%)		
2	51 (98.08%)	9 (100.00%)	42 (97.67%)		
Historyofglucocorticoiduse (1yes,2no)					>0.999
1	1 (1.92%)	0 (0.00%)	1 (2.33%)		
2	51 (98.08%)	9 (100.00%)	42 (97.67%)		
Tumor(1yes,2no)					0.670
1	10 (19.23%)	1 (11.11%)	9 (20.93%)		
2	42 (80.77%)	8 (88.89%)	34 (79.07%)		
Hematologicalsystemdiseases(1yes,2no)					>0.999
1	1 (1.92%)	0 (0.00%)	1 (2.33%)		
2	51 (98.08%)	9 (100.00%)	42 (97.67%)		
^1^Median [IQR]; n (%)					
^2^Wilcoxon rank sum test; Fisher’s exact test					

BMI, Body Mass Index; COPD, Chronic Obstructive Pulmonary Disease; CKD, Chronic Kidney Disease.

Kaplan–Meier survival analysis demonstrated that sepsis patients with high plasma levels of PSMA7 or PSMB2 had significantly lower 90–day survival rates than those with low expression (Log–rank test, *P* < 0.05; **Figures 3D, E**). In contrast, PSMB3 expression showed no statistical association with survival (Log–rank test, *P* = 0.39; **Supplementary Figure 1**). After adjusting for age and SOFA score, both remained independent risk factors (PSMA7: HR = 1.93, 95% CI: 1.14–3.26, *P* = 0.014; PSMB2: HR = 1.93, 95% CI: 1.24–2.99, *P* = 0.004; **Figure 3F**).

To further validate the clinical relevance of PSMA7 and PSMB2 at the transcriptional level, we performed a meta-analysis of 10 independent GEO datasets (total n = 1,174; 307 healthy controls, 867 sepsis patients). The results are summarized in **Figures 3G, H**, **Supplementary Tables 1**, **S2**. Compared with healthy controls, PSMA7 mRNA levels showed no significant difference in sepsis patients overall (SMD = -0.40, 95% CI: -0.90 – 0.09, *P* = 0.113), with high heterogeneity (I² = 90%, τ² = 0.55, Q-test *P* < 0.01). Leave-one-out analysis revealed that the non-significant result was sensitive to the inclusion of two studies (GSE236713 and GSE100159); removal of either study yielded a significant positive association (*P* < 0.05), suggesting that the overall null finding is not robust and may depend on specific cohorts. No significant publication bias was detected (Egger’s test *P* = 0.47).Among sepsis patients, non-survivors exhibited significantly higher PSMA7 expression than survivors (SMD = -0.42, 95% CI: -0.68 – -0.16, *P* = 0.001), with no heterogeneity (I² = 0%, τ² = 0, Q-test *P* = 0.95). The result remained significant after excluding any single study, except when GSE185263 was removed (*P* = 0.08), indicating a modest dependency on this large study. Egger’s test showed no asymmetry (*P* = 0.63, based on 3 studies).

PSMB2 mRNA was significantly downregulated in sepsis patients compared to healthy controls (SMD = 0.65, 95% CI: 0.21 – 1.10, *P* = 0.004), with high heterogeneity (I² = 88%, τ² = 0.43, Q-test *P* < 0.01). The positive SMD indicates lower expression in sepsis patients relative to controls. Leave-one-out analysis confirmed the robustness of this finding: the pooled estimate remained significant (*P* < 0.05) after removal of any single study, with SMD values ranging from 0.49 – 0.79. No significant publication bias was observed (Egger’s test *P* = 0.76). In the survivor vs non-survivor comparison, PSMB2 expression was marginally higher in non-survivors (SMD = -0.58, 95% CI: -1.16 – 0.001, *P* = 0.0504), with moderate heterogeneity (I² = 67%, τ² = 0.17, Q-test *P* = 0.05). Sensitivity analysis revealed that this result was strongly influenced by GSE185263: after excluding this study, the association became highly significant (SMD ≈ -0.90, *P* < 0.001) with reduced heterogeneity, whereas removal of other studies yielded non-significant P-values. Egger’s test suggested no significant asymmetry (*P* = 0.09), though interpretation is limited by the small number of studies.

To investigate the correlation of PSMA7 and PSMB2 protein levels with organ dysfunction, we performed correlation analysis combining proteomic data with clinical indicators. Both PSMA7 and PSMB2 levels showed significant associations with key clinical parameters. Each correlated strongly with markers of hepatocellular injury (AST, ALT; e.g., PSMA7 vs. AST: Pearson r = 0.573, *P* = 5.86×10^-5^). Notably, PSMB2 level positively correlated with disease severity (SOFA score: r = 0.436, *P* = 0.004). PSMA7 also positively correlated with systemic inflammatory markers (e.g., WBC, NEUT). Additionally, PSMA7 was negatively associated with platelet count (Plt), while PSMB2 was negatively correlated with glomerular filtration rate (GFR) (**Figure 3I**).

### 3.4 Molecular docking and dynamics simulations suggest stable binding of Handelin to PSMA7/PSMB2

**Figure 4:**
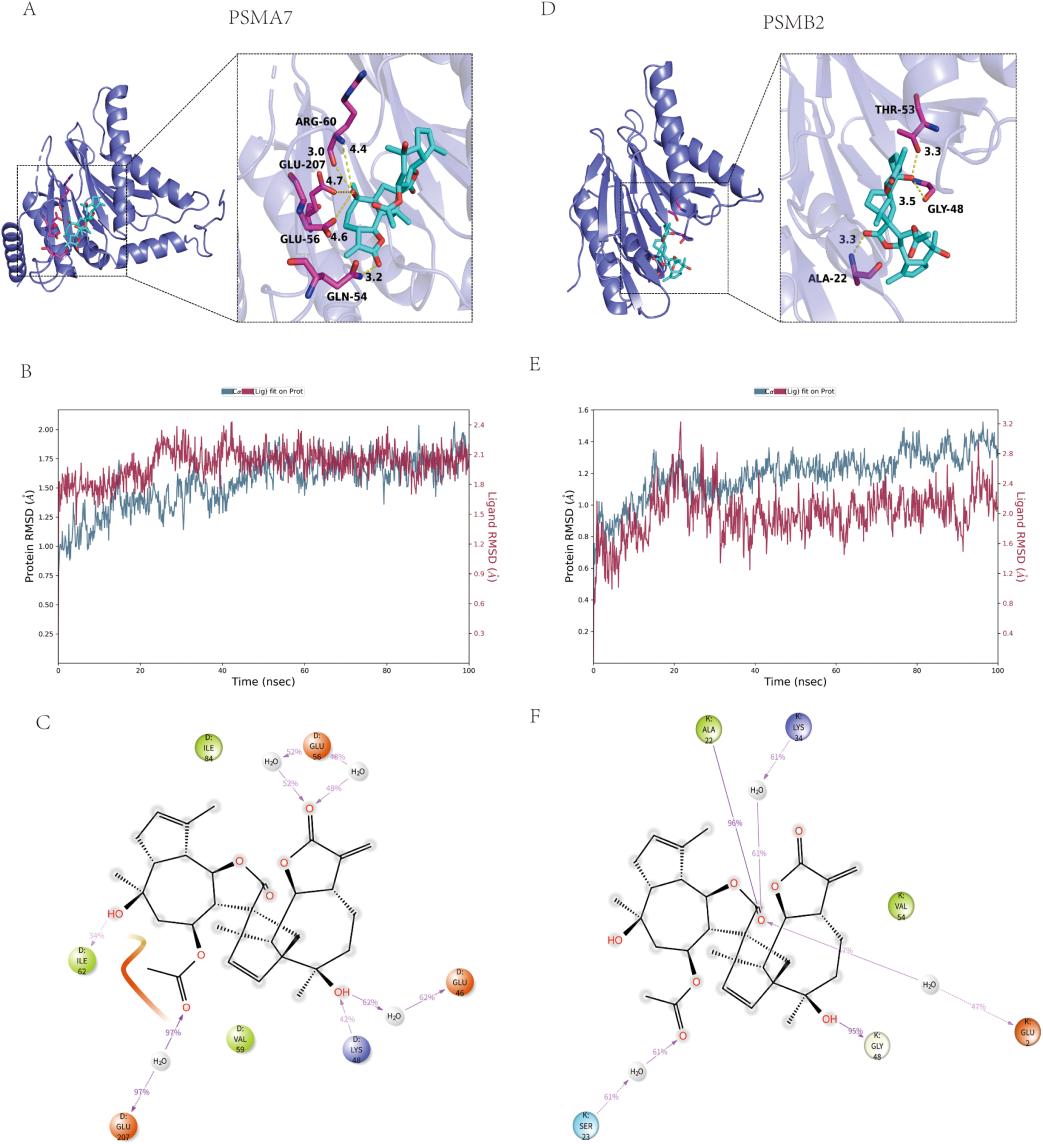
Computational analysis of Handelin binding to PSMA7 and PSMB2. **(A)** Molecular docking pose of Handelin (cyan sticks) within the PSMA7 binding pocket (dark blue cartoon). Key interacting residues are shown in magenta sticks. Non-polar hydrogen atoms are omitted. The predicted binding free energy is -7.6 kcal/mol. **(B)** Two-dimensional interaction map of the Handelin-PSMA7 complex. Hydrogen bonds, ionic interactions, and hydrophobic interactions are depicted as yellow, magenta, and green dashed lines, respectively. Key residues and interaction distances (Å) are labeled. **(C)** Root-mean-square deviation (RMSD) analysis from the 100-ns molecular dynamics simulation of the PSMA7-Handelin complex. The RMSD of the PSMA7 protein backbone (top) stabilizes within 1.5–2.0 Å, and the RMSD of the Handelin ligand (bottom) stabilizes at ~2.1 (Å) **(D)** Molecular docking pose of Handelin (cyan sticks) within the PSMB2 binding pocket (dark blue cartoon). Key interacting residues are shown in magenta sticks. Non-polar hydrogen atoms are omitted. The predicted binding free energy is -8.0 kcal/mol. **(E)** Two-dimensional interaction map of the Handelin-PSMB2 complex. A notable dual-hydrogen-bond network with the catalytic site residue Thr53 is highlighted. Other annotations are as in panel **(B, F)** Root-mean-square deviation (RMSD) analysis from the 100-ns molecular dynamics simulation of the PSMB2-Handelin complex. The RMSD of the PSMB2 protein backbone (top) stabilizes within 1.0–1.6 Å, and the RMSD of the Handelin ligand (bottom) stabilizes at ~2.2 Å.

To explore the binding mechanism between Handelin and the key target proteins at the atomic level, we performed computational simulations. Molecular docking revealed that Handelin could dock into potential binding pockets of PSMA7 (**Figure 4A**) and PSMB2 (**Figure 4D**) with favorable predicted binding free energies of –7.6 kcal/mol and –8.0 kcal/mol, respectively. For PSMA7, the selected pocket was a predicted allosteric cavity at the intersubunit interface of the α-ring, proximal to the gate region controlling substrate entry. For PSMB2, docking was centered on the trypsin-like catalytic site to assess potential interactions with the active center, with Handelin forming a stable dual-hydrogen-bond network with Thr53. Detailed interaction analysis showed that Handelin formed distinct yet robust binding networks with both target proteins (**Figures 4B, E**). For PSMA7, Handelin primarily formed hydrogen bonds with residues such as Gln54 and Arg60 (distances 3.2 Å and 3.0 Å, respectively) and was buried in a hydrophobic pocket formed by Ile62, Val59, Ile84, etc., with the hydrophobic interaction with Ile62 maintained over 97% of the simulation time (**Figure 4B**). For PSMB2, a notable finding was the formation of a stable dual–hydrogen–bond network with Thr53, a residue near the catalytic site (distances 3.3 Å and 3.5 Å, occupancy 61% and 95%, respectively), along with extensive hydrophobic contacts with Ala22, Val54, and others (**Figure 4E**).

Molecular dynamics simulations over 100 ns confirmed the stability of the complexes (**Figures 4C, F**). The backbone RMSD of PSMA7 and PSMB2 remained stable, converging within 1.5–2.0 Å and 1.0–1.6 Å, respectively. Importantly, the ligand RMSD of Handelin in its binding pocket converged and stabilized at approximately 2.1 Å (PSMA7; **Figure 4C**) and 2.2 Å (PSMB2; **Figure 4F**) after initial equilibration, indicating limited positional fluctuation and formation of a dynamically stable complex.

## 4 Discussion

This study demonstrates that the natural compound Handelin exerts protective effects in a zebrafish sepsis-like model, including attenuation of oxidative stress, reduction of macrophage activation, and improved survival. The findings suggest that its core mechanism may involve direct binding and stabilization of key 26S proteasome subunits, PSMA7 and PSMB2. Clinical analysis further indicates that higher plasma levels of PSMA7 and PSMB2 are significantly associated with increased 90-day mortality in sepsis patients, despite complex expression dynamics during disease progression. These discoveries link Handelin, proteasome function, and sepsis prognosis, providing not only a novel perspective for understanding the role of the proteasome in sepsis but also revealing a new potential therapeutic target.

Handelin has previously been shown to maintain proteostasis in models of muscle atrophy and aging by modulating protein degradation pathways (8, 17). Extending these findings, our study demonstrates that Handelin confers protection in a sepsis-like model, reducing mortality, inflammation, and oxidative stress (**Figure 1**). These observations, together with the identification of proteasome subunits as Handelin-interacting proteins, suggest that Handelin may exert its broad protective effects by targeting core components of the cellular protein quality control network.

To identify potential protein targets of Handelin, we performed an unbiased screening using the DIA-CETSA technique in a macrophage model. The macrophage was chosen as the screening system because these cells are central drivers of immune dysregulation in sepsis (18, 19), and the innate immune system of zebrafish larvae is highly conserved (20). The screening results revealed the “proteasome” pathway as the most significantly enriched among proteins with markedly increased thermal stability upon Handelin treatment (**Figure 2**). Molecular docking and dynamics simulations further support that Handelin can stably bind to the core proteasome subunits PSMA7 and PSMB2 (**Figure 4**). These findings provide the first evidence linking Handelin’s biological effects to the proteasome, offering a new molecular basis for explaining its pleiotropic actions.

Recent advances in sepsis biomarker discovery have leveraged multi-omics integration and machine learning to identify immune cell-specific signatures (21–23). Our study offers a distinct and complementary perspective: rather than identifying a diagnostic or prognostic signature, we have pinpointed a druggable protein complex—the 26S proteasome—whose pharmacological modulation by Handelin confers functional protection *in vivo*. This moves beyond association-based biomarkers toward a direct therapeutic link.

Clinical proteomics revealed that elevated plasma levels of PSMA7 and PSMB2 were significantly associated with increased 90-day mortality (**Figures 3D, E**), and both proteins remained independent risk factors after adjusting for age and SOFA score (**Figure 3F**). Furthermore, PSMA7 and PSMB2 levels correlated positively with markers of liver injury (AST, ALT) and disease severity (SOFA score) (**Figure 3I**). These findings suggest that upregulation of these proteasome subunits reflects a state of proteasome hyperactivation and dysregulation that contributes to organ dysfunction and poor outcomes in sepsis.

The integration of transcriptomic meta-analysis with our plasma proteomic data revealed intriguing discrepancies. While PSMA7 protein was elevated in non-survivors, its mRNA showed no consistent difference between sepsis and controls. Conversely, PSMB2 mRNA was significantly downregulated in sepsis patients, yet its protein levels were elevated. The discordance between PSMB2 mRNA and protein provides direct evidence for enhanced post-transcriptional stabilization of this subunit under inflammatory stress. These observations underscore the importance of orthogonal protein-level validation and suggest that post-transcriptional mechanisms play a major role in determining proteasome subunit abundance during sepsis.

An apparent paradox emerges when comparing the clinical and mechanistic findings: elevated PSMA7/PSMB2 levels are associated with poor outcomes, yet Handelin-induced stabilization of these subunits is associated with protective effects *in vivo*. We propose that this paradox can be resolved by distinguishing between quantitative expression and qualitative functional states. Elevated PSMA7/PSMB2 in sepsis patients likely reflects a state of proteasome hyperactivation and dysregulation—a “more but worse” scenario in which increased proteasome capacity is coupled with loss of substrate fidelity, leading to excessive degradation of regulatory proteins (e.g., IκBα) and structural proteins. In contrast, Handelin-induced thermal stabilization may represent a qualitatively different phenomenon: rather than simply increasing protein abundance, Handelin may act as an allosteric modulator, stabilizing a conformation of PSMA7/PSMB2 that favors proper substrate recognition over promiscuous degradation. This would preserve essential quality control functions while limiting pathological over-degradation. The molecular docking results support this possibility, revealing that Handelin binds near the catalytic site of PSMB2 (Thr53) and forms stable interactions that could influence substrate access or processing. Thus, Handelin may improve the “quality” rather than the “quantity” of proteasome function—a framework that reconciles the clinical observation (elevated levels as a marker of stress and dysregulation) with the mechanistic finding (stabilization as a marker of functional optimization).

The conventional view holds that the 26S proteasome plays a dual role in sepsis (24–27). On one hand, it acts as an “inflammatory amplifier” and “catabolic executor”: elevated activity drives muscle wasting via NF-κB/FOXO pathways (28), exacerbates cytokine storms by controlling IκBα turnover (29), and parallels organ dysfunction (25). On the other hand, the proteasome is indispensable as a “quality controller”, clearing misfolded proteins and supporting antigen presentation (26). Complete inhibition may compromise host defense (26), leading to interest in strategies for precise modulation rather than global inhibition (25, 27).

Within this framework, a potential mechanism of Handelin’s action can be considered. Our CETSA results indicate that Handelin treatment is associated with increased thermal stability of core proteasome subunits. Based on these findings, we speculate that Handelin may facilitate a more balanced functional state of the proteasome under septic stress. Specifically, by stabilizing the core subunits, Handelin may help preserve structural integrity and substrate recognition specificity, potentially allowing essential quality control while limiting harmful over-degradation. Coupled with its known effect of activating Hsp70 (17), Handelin may contribute to the overall cellular protein quality control network, offering a plausible unifying hypothesis for its broad protective effects. It is important to note, however, that this proposed mechanism remains inferential at this stage, as direct measurements of proteasome activity and substrate profiles after Handelin treatment were not performed in this study.

This study has several limitations. First, the clinical data are cross-sectional; dynamic validation in time-series animal models is warranted. Future studies should verify the necessity of PSMA7/PSMB2 in Handelin’s effects through genetic manipulations in cellular and animal models. Second, techniques such as quantitative degradomics could directly test Handelin’s effects on proteasome substrate profiles. Third, the therapeutic potential of Handelin requires evaluation in higher-order animal models (e.g., murine CLP model). Fourth, while CETSA suggests interaction between Handelin and PSMA7/PSMB2, direct binding affinity and kinetics require validation using purified proteins and surface plasmon resonance. The functional consequences of this interaction—whether it enhances, inhibits, or qualitatively alters proteasome activity—remain to be determined. The proposed “functional optimization” hypothesis, while mechanistically plausible, requires direct testing using *in vitro* proteasome activity assays and cellular models with genetic manipulation of target subunits.

Several limitations of the meta-analysis should also be acknowledged. Moderate to high heterogeneity was observed in the comparisons between healthy controls and sepsis patients for both PSMA7 and PSMB2, likely attributable to differences in patient demographics, disease severity, sampling time, and microarray platforms across studies. However, the consistent direction of effects (except for PSMA7’s overall null result) and the robustness demonstrated by leave-one-out analyses (particularly for PSMB2) suggest that the main conclusions are reliable. The survivor vs. non-survivor comparisons included only three studies, limiting the power of Egger’s test and increasing the influence of individual studies, as evidenced by the sensitivity of PSMB2 results to GSE185263. These findings should therefore be interpreted cautiously and warrant validation in larger, harmonized cohorts.

## 5 Conclusions

In summary, this study integrates chemoproteomic target identification, clinical proteomics, and transcriptomic meta-analysis to reveal that the protective effects of Handelin in sepsis may be mediated through its interaction with the core proteasome subunits PSMA7 and PSMB2. Our clinical findings demonstrate that elevated plasma levels of these subunits are independently associated with increased mortality, suggesting that proteasome dysregulation—rather than simply increased abundance—contributes to poor outcomes in sepsis. This apparent paradox with Handelin’s protective stabilization of the same subunits is reconciled by distinguishing between quantitative overexpression (a marker of pathological hyperactivation) and qualitative functional optimization (allosteric modulation toward substrate fidelity). We propose that Handelin may facilitate this functional rebalancing, preserving essential quality control while limiting harmful over-degradation. This discovery not only provides a new molecular basis for the potential anti-sepsis application of Handelin but also suggests a therapeutic strategy centered on precision modulation of proteasome function—an approach that aligns with emerging concepts in the field and offers an alternative to global proteasome inhibition.

## Data Availability

The DIA-CETSA mass spectrometry proteomics data generated in this study are available from the corresponding author upon reasonable request. The human plasma proteomics data have been deposited in the OMIX repository under accession code OMIX014169 (https://ngdc.cncb.ac.cn/omix/select-edit/OMIX014169). Relevant clinical data are available from the corresponding author upon reasonable request.
