# Supplementary material for: Multi-omic profiling converges on proteasome subunits PSMA7/PSMB2 as targets of the sepsis-protective agent Handelin
Source: Front Immunol. 2026 Mar 19;17:1782122. doi: 10.3389/fimmu.2026.1782122 (PMC13043404; doi:10.3389/fimmu.2026.1782122)
Supplement: Supplementary file 1 [file Supplementaryfile1.docx]

**Table S1. Leave-One-Out Sensitivity Analysis for PSMA7 and PSMB2 Meta-Analyses**

1. PSMA7: Control vs Sepsis

| **Omitted Study** | **SMD** | **CI_lower** | **CI_upper** | **pval** | **I² (%)** |
| --- | --- | --- | --- | --- | --- |
| None (All studies) | -0.4010 | -0.8964 | 0.0944 | 0.1127 | 90.27 |
| Without GSE163151 | -0.3641 | -0.8905 | 0.1624 | 0.1753 | 91.32 |
| Without GSE232753 | -0.4680 | -0.9883 | 0.0522 | 0.0778 | 90.96 |
| Without GSE95233 | -0.2611 | -0.7535 | 0.2313 | 0.2987 | 88.99 |
| Without GSE69063 | -0.4276 | -0.9688 | 0.1136 | 0.1215 | 91.17 |
| Without GSE243217 | -0.4467 | -0.9780 | 0.0845 | 0.0993 | 91.06 |
| Without GSE236713 | -0.5002 | -0.9949 | -0.0055 | 0.0475 | 88.40 |
| Without GSE185263 | -0.4172 | -0.9985 | 0.1640 | 0.1594 | 90.93 |
| Without GSE154918 | -0.2834 | -0.7968 | 0.2301 | 0.2794 | 89.87 |
| Without GSE134347 | -0.3128 | -0.8482 | 0.2225 | 0.2521 | 88.84 |
| Without GSE100159 | -0.5262 | -1.0209 | -0.0316 | 0.0370 | 89.69 |

2. PSMA7: Survivor vs Non-Survivor

| **Omitted Study** | **SMD** | **CI_lower** | **CI_upper** | **pval** | **I² (%)** |
| --- | --- | --- | --- | --- | --- |
| None (All studies) | -0.4245 | -0.6845 | -0.1646 | 0.0014 | 0 |
| Without GSE33118 | -0.4154 | -0.6802 | -0.1506 | 0.0021 | 0 |
| Without GSE95233 | -0.4354 | -0.7171 | -0.1538 | 0.0024 | 0 |
| Without GSE185263 | -0.4385 | -0.9299 | 0.0530 | 0.0804 | 0 |

3. PSMB2: Control vs Sepsis

| **Omitted Study** | **SMD** | **CI_lower** | **CI_upper** | **pval** | **I² (%)** |
| --- | --- | --- | --- | --- | --- |
| None (All studies) | 0.6539 | 0.2056 | 1.1022 | 0.0043 | 87.88 |
| Without GSE163151 | 0.7888 | 0.3508 | 1.2267 | 0.0004 | 87.17 |
| Without GSE232753 | 0.6037 | 0.1291 | 1.0783 | 0.0127 | 89.04 |
| Without GSE95233 | 0.4878 | 0.1045 | 0.8710 | 0.0126 | 81.37 |
| Without GSE69063 | 0.6999 | 0.2142 | 1.1856 | 0.0047 | 88.81 |
| Without GSE243217 | 0.6077 | 0.1258 | 1.0897 | 0.0134 | 89.05 |
| Without GSE236713 | 0.6687 | 0.1569 | 1.1805 | 0.0104 | 89.08 |
| Without GSE185263 | 0.7130 | 0.2243 | 1.2017 | 0.0042 | 86.85 |
| Without GSE154918 | 0.6996 | 0.2054 | 1.1937 | 0.0055 | 88.57 |
| Without GSE134347 | 0.5800 | 0.0966 | 1.0634 | 0.0187 | 86.11 |
| Without GSE100159 | 0.6833 | 0.1986 | 1.1680 | 0.0057 | 89.08 |

4. PSMB2: Survivor vs Non-Survivor

| **Omitted Study** | **SMD** | **CI_lower** | **CI_upper** | **pval** | **I² (%)** |
| --- | --- | --- | --- | --- | --- |
| None (All studies) | -0.5794 | -1.1600 | 0.0011 | 0.0504 | 66.68 |
| Without GSE33118 | -0.4051 | -0.9720 | 0.1618 | 0.1614 | 66.49 |
| Without GSE95233 | -0.5614 | -1.5194 | 0.3967 | 0.2508 | 73.56 |
| Without GSE185263 | -0.8763 | -1.3873 | -0.3654 | 0.0008 | 0 |

**Table S2. Egger's Test for Publication Bias**

| **Gene** | **Comparison** | **Studies**  **(n)** | **t-value** | **df** | **P-value** | **Interpretation** |
| --- | --- | --- | --- | --- | --- | --- |
| PSMA7 | Control vs Sepsis | 10 | 0.7613 | 8 | 0.4683 | No significant asymmetry |
| PSMA7 | Survivor vs Non-survivor | 3 | -0.6647 | 1 | 0.6266 | No significant asymmetry* |
| PSMB2 | Control vs Sepsis | 10 | 0.32 | 8 | 0.76 | No significant asymmetry |
| PSMB2 | Survivor vs Non-survivor | 3 | -7.1430 | 1 | 0.0885 | No significant asymmetry* |

*Note: Egger's test has low power when number of studies < 10. Results should be interpreted with caution.

Figure 1


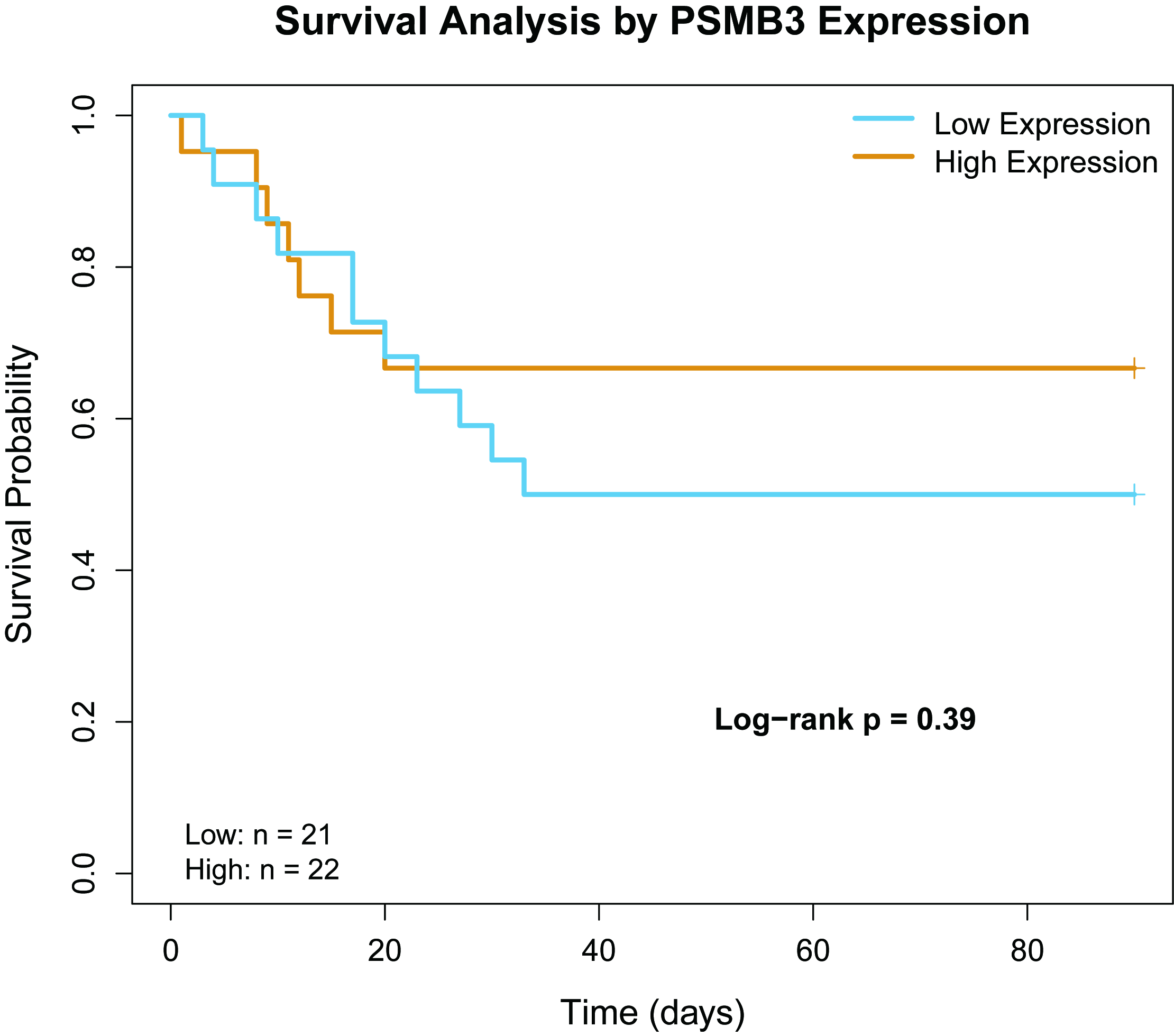


###### Determination of the 72 hpf LC50 of Lipopolysaccharide in Zebrafish Larvae

**Principle and Methods**

This study aimed to determine the median lethal concentration (LC50) of lipopolysaccharide (LPS) in zebrafish larvae.Bacterial endotoxin, also known as lipopolysaccharide (LPS), is a component of the cell wall of Gram-negative bacteria. It can induce fever, immune dysfunction, microcirculation disorders, and multi-organ inflammation in animals, making it suitable for establishing zebrafish models of inflammation.In the first part of the study, zebrafish embryos at 6 hours post-fertilization (hpf) were exposed to test samples. Each group consisted of 10 samples with three replicates. Dead embryos were removed and the solution was renewed every 24 hours. At 72 hpf, heart rate, mortality rate, malformation rate, and types of malformations were assessed to determine the LC50 at 72 hpf.

**Experimental System**

Test system: Wild-type AB strain zebrafish，Zebrafish age: 6 hours post-fertilization (6 hpf)，Sample size per group: 10 larvae.

**Instruments and Equipment**

Laboratory instruments:Artificial climate incubator (RGX-70ES)，Vortex mixer (ZD-85)，Water bath, etc.

Laboratory consumables:90 mm Petri dishes,6-well plates,3 mL plastic droppers,15 mL centrifuge tubes, etc.

**Experimental Procedure**

Drug solution preparation:After sample collection, 20 mg of the sample was weighed, dissolved in 1 mL of E3 medium, heated to dissolve, and mixed by vortexing to obtain a 20 mg/mL stock solution.Aliquots of 200, 100, 50, and 25 μL of this stock solution were added to 50 mL of E3 solution and mixed by vortexing to prepare the experimental exposure concentrations of 80, 40, 20, and 10 μg/mL, respectively.

Exposure of 6 hpf zebrafish:150 healthy 6 hpf wild-type (WT) AB strain zebrafish were selected and randomly divided into a control group and four exposure groups (at the concentrations listed above), with 10 fish per group and three replicates.Larvae in the exposure groups were exposed to the corresponding drug solutions until 72 hpf, with the solution renewed every 24 hours.At 72 hpf, heart rate, mortality rate, malformation rate, and types of malformations were recorded under a microscope.

**Results**


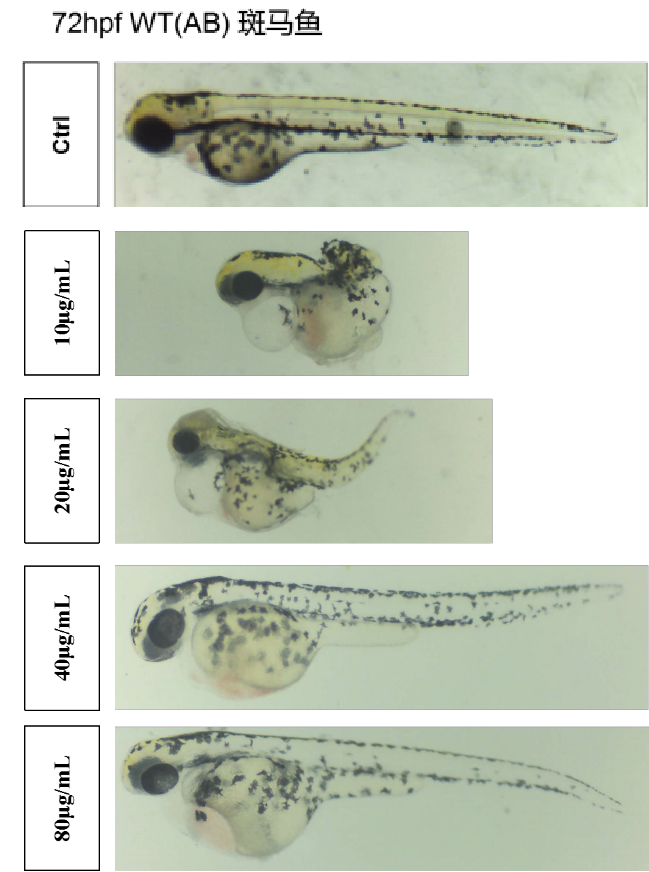


****Figure 2. Development of zebrafish larvae at 72 hpf****


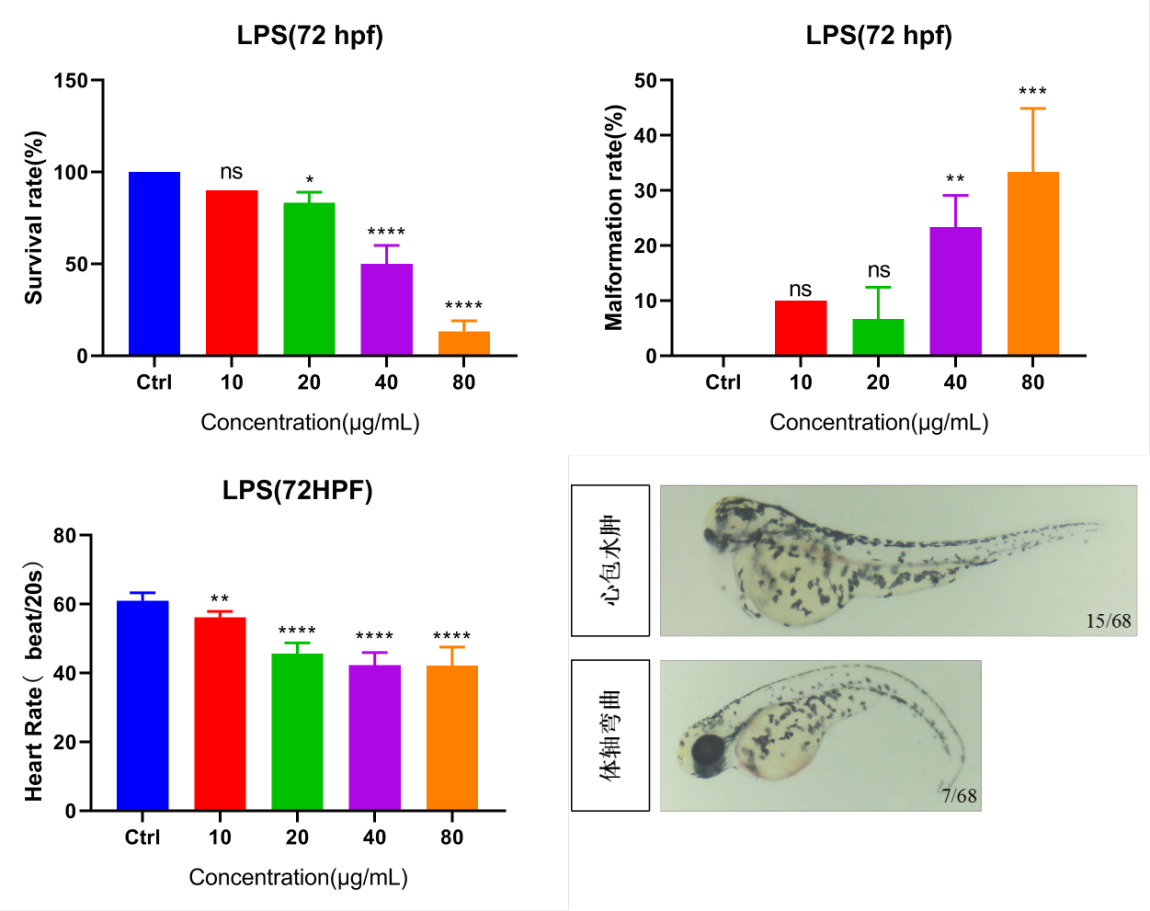


****Figure3. Survival rate, heart rate, and malformation rate of zebrafish larvae at 72 hpf****


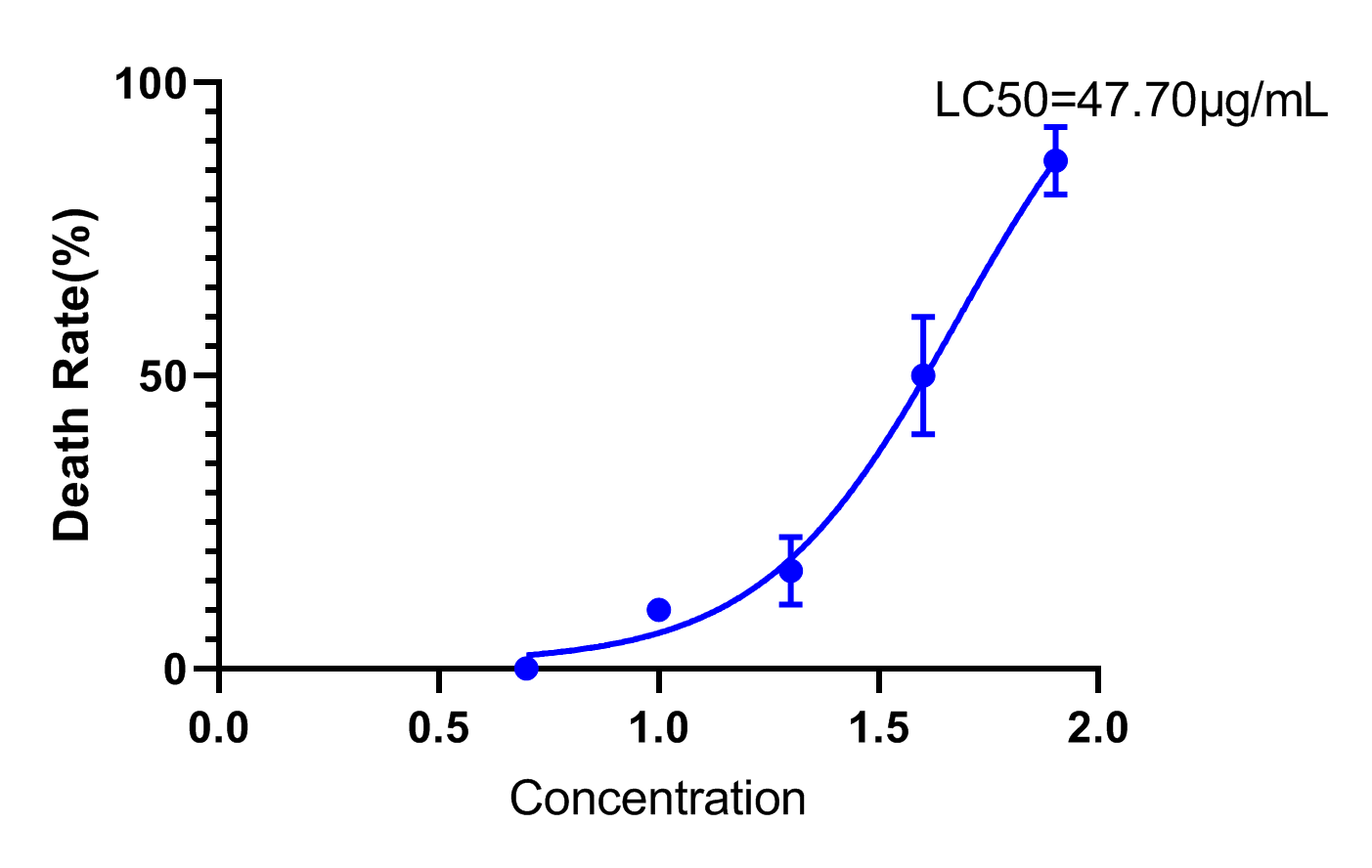


****Figure4. LC50 curve for zebrafish larvae exposed to LPS for 72 hpf****

****Result:** The LC50 of lipopolysaccharide at 72 hpf was calculated by non-linear fitting to be **47.70 μg/mL**.**
